# Identifying patients with Lynch syndrome using a universal tumor screening program in an integrated healthcare system

**DOI:** 10.1186/s13053-022-00217-1

**Published:** 2022-04-18

**Authors:** Philip R. Crain, Jamilyn M. Zepp, Sara Gille, Lindsay Jenkins, Tia L. Kauffman, Elizabeth Shuster, Katrina A.B. Goddard, Benjamin S. Wilfond, Jessica Ezzell Hunter

**Affiliations:** 1 Department of Biostatistics, School of Public Health, University of Washington, Seattle, WA USA; 2 Department of Translational and Applied Genomics, Center for Health Research, Kaiser Permanente Northwest, Portland, OR USA; 3 Center for Health Research, Kaiser Permanente Northwest, Portland, OR USA; 4 Treuman Katz Center for Pediatric Bioethics, Department of Pediatrics, Seattle Children’s Research Institute and Hospital, University of Washington School of Medicine, Seattle, WA USA

**Keywords:** Lynch syndrome, HNPCC, Colorectal cancer, Genetic screening, Genetic testing, Microsatellite instability

## Abstract

**Introduction:**

Lynch syndrome (LS) is associated with an increased risk of colorectal (CRC) and endometrial (EC) cancers. Universal tumor screening (UTS) of all individuals diagnosed with CRC and EC is recommended to increase identification of LS. Kaiser Permanente Northwest (KPNW) implemented a UTS program for LS among individuals newly diagnosed with CRC in January 2016 and EC in November 2016. UTS at KPNW begins with immunohistochemistry (IHC) of tumor tissue to determine loss of mismatch repair proteins associated with LS (MLH1, MSH2, MSH6, and PMS2)., IHC showing loss of MLH1 is followed by reflex testing (automatic testing) to detect the presence of the *BRAF* V600E variant (in cases of CRC) and *MLH1* promoter hypermethylation to rule out likely sporadic cases.

**Materials and methods:**

Individuals newly diagnosed with CRC and EC were identified between the initiation of the respective UTS programs and July 2018. Electronic medical records were reviewed to extract patient data related to UTS, including IHC and reflex testing results, date of referrals to the genetics department, and results of germline genetic testing for LS.

**Results:**

313 out of 362 individuals diagnosed with CRC and 61 out of 64 individuals diagnosed with EC who were eligible were screened by IHC for LS. Most (47/52 or 90%, including 46/49 CRC and 1/3 EC) individuals that were not screened by IHC only had a biopsy sample available. Fourteen individuals (3.7% overall, including 13/313 CRC and 1/61 EC) received an abnormal result after reflex testing and were referred for genetic counseling. Of these, 10 individuals (71% overall, including 9/13 CRC and 1/1 EC) underwent germline genetic testing for LS. Five individuals diagnosed with CRC were found to have pathogenic variants. in *PMS2* (*n* = 3), *MLH1* (*n* = 1), and *MSH6* (*n* = 1). No pathogenic variants were identified in individuals diagnosed with EC.

**Conclusions:**

UTS identified individuals at risk for LS. Most individuals who screened positive for LS had follow-up germline genetic testing for LS. The consistent use of biopsy samples is an opportunity to improve UTS.

## Introduction

Lynch syndrome (LS) is a hereditary cancer syndrome which notably leads to an increased risk of colorectal cancer (CRC) and endometrial cancer (EC), among many other cancer types. Males diagnosed with LS have a lifetime risk of 30–75% for CRC, while females diagnosed with LS face a lifetime risk of 25–50% for CRC and 30–40% for EC. [1] LS accounts for 3% of all cases of CRC and 2% of all cases of EC. [2–4] This autosomal dominant condition is caused by pathogenic variants in DNA mismatch repair (MMR) genes (*MLH1*, *MSH2*, *MSH6*, or *PMS2*) [5, 6] and deletions in *EPCAM* which result in inactivation of *MSH2*. [7, 8] Despite a prevalence estimate of 1 in 279 for LS in the United States general population, less than 2% of people affected by LS have been diagnosed. [9, 10] Undiagnosed individuals represent a missed opportunity to implement medical interventions to reduce cancer-related morbidity and mortality, including increased surveillance and risk-reducing surgery or medication. [1, 11, 12]

One approach to identify cases of LS focuses on the presence of high-risk criteria, such as a personal and family history, or early age at onset, of LS-associated cancers. [13] The Revised Amsterdam and Bethesda criterias [14–17] and risk assessment algorithms, such as PREMM_5_, MMRpro, and MMRpredict, [18–20] are examples of this approach to identify individuals appropriate for germline genetic testing for LS. However, this approach will miss LS in individuals who have limited or unreliable family history, do not meet the high-risk criteria, [21, 22] or meet the high-risk criteria but do not undergo genetic counseling and germline genetic testing for LS. [23–28] Universal tumor screening (UTS) for LS is a different approach to identifying individuals at heightened risk for LS. This approach includes all individuals newly diagnosed with CRC and EC, regardless of family history or age. Identification of individuals at heightened risk based upon cancer diagnosis as opposed to other risk criteria may lead to LS diagnoses in individuals who would otherwise be missed. UTS is recommended by multiple expert guidelines to increase the identification of LS. [13, 29–34]. Though the exact approach for UTS may vary across health care systems, the first step is typically screening tumor tissue with a test for, (microsatellite instability (MSI) or with immunohistochemistry (IHC) testing) to detect the absence of one or more of the MMR proteins (MLH1, MSH2, MSH6, and PMS2). Reflex testing follows if MLH1 loss is reported to detect the presence of *BRAF* V600E or *MLH1* promoter hypermethylation, two indicators of sporadic *MLH1* loss unrelated to LS. Individuals that screen positive for LS (loss of one or more of the MMR proteins is detected and sporadic indicators are not found) after these steps are then referred for genetic counseling and germline genetic testing to confirm an LS diagnosis. [4]

UTS has been implemented into many healthcare systems, but its uptake is still limited. Many barriers exist for healthcare systems to implement effective UTS programs. [35–37] Additionally, there are limited data on the outcomes of UTS in community healthcare settings, including measures of uptake of genetic counseling and germline genetic testing for LS. While there is substantial evidence to support UTS as valuable for identifying cases of LS, mixed results have been reported even in healthcare systems and regions similar to Kaiser Permanente Northwest (KPNW). [38, 39]

The goal of this study was to assess the effectiveness of a UTS program implemented by an integrated healthcare system by systematically determining whether all tumors from individuals with CRC and EC that should be screened with IHC were screened and to assess the rate of appropriate follow-up with the genetics department. This study adds to the evidence about UTS by detailing the flow of patients through the UTS process to identify points in the process that could be improved to maximize the effectiveness of UTS in identifying individuals with LS.

## Materials and methods

### Study population

KPNW serves more than 625,000 of the nearly 3,400,000 people in northwest Oregon and southwest Washington. KPNW is an integrated health care system, which is a membership-based, prepaid, direct health care system where members have access to care and services that are coordinated across inpatient and outpatient settings, pharmacy, lab, imaging, and other ancillary services. KPNW began to perform UTS among all individuals newly diagnosed with CRC in January 2016 and all individuals newly diagnosed with EC in November 2016.

### Universal tumor screening protocol

According to the UTS protocol at KPNW, each CRC and EC is tested by immunohistochemistry (IHC) to detect the presence or absence of the 4 MMR proteins: MLH1, MSH2, MSH6, or PMS2. If loss of MLH1 is detected, reflex testing is performed. For CRC tumors, reflex testing starts with testing for the presence of *BRAF* V6000E, followed by testing for *MLH1* hypermethylation if *BRAF* V600E is absent. For EC tumors, reflex testing consists of testing for *MLH1* hypermethylation only. The presence of *BRAF* V600E or *MLH1* hypermethylation indicate likely non-inherited (sporadic) tumor development, meaning an LS diagnosis in these individuals is atypical and genetic counseling and germline genetic testing for LS is not indicated. Individuals with MMR loss detected through IHC, and absence of *BRAF* V600E and/or *MLH1* hypermethylation for individuals with detected MLH1 loss, are referred to the genetics department for follow-up. Patients meet with a genetic counselor to discuss LS, their risk, and diagnostic testing is ordered, if indicated. Germline genetic testing is typically performed using a comprehensive panel of cancer risk genes. Test results are disclosed by the genetic counselor.

### EMR review

The KPNW Tumor Registry was searched for all diagnoses of CRC (ICD-10 diagnosis codes: C18.0, C18.2, C18.3, C18.4, C18.5, C18.6, C18.7, C18.8, C18.9, C19.9, C20.9, and C21.8) and EC (ICD-10 diagnosis code: C54.1) between 2016 and July 2018. We excluded individuals who opted to have their medical data excluded from all types of research and individuals who opted to be excluded from genetics research. Electronic medical record (EMR) review of each case was performed by a study genetic counselor (JZ) to extract patient data related to UTS: date of birth, date of biopsy and/or surgery, results of IHC performed on tumor tissue, results of reflex testing, date of referrals to genetics department, date of encounters with genetics department, date germline genetic testing ordered, and results of germline genetic testing. For patients who underwent germline genetic testing and received a diagnosis of LS, notes captured during the genetic counseling session on personal and family history as well as pathology reports were used to assess whether the patient met Bethesda Guidelines [16, 17] or Amsterdam criteria [14, 15]. This analysis was approved by the Institutional Review Board at KPNW.

### Statistical analysis

Detection rates for LS in CRC samples were calculated along with the associated 95% Wilson score confidence intervals.

## Results

### Universal tumor screening in CRC patients

**Fig. 1:**
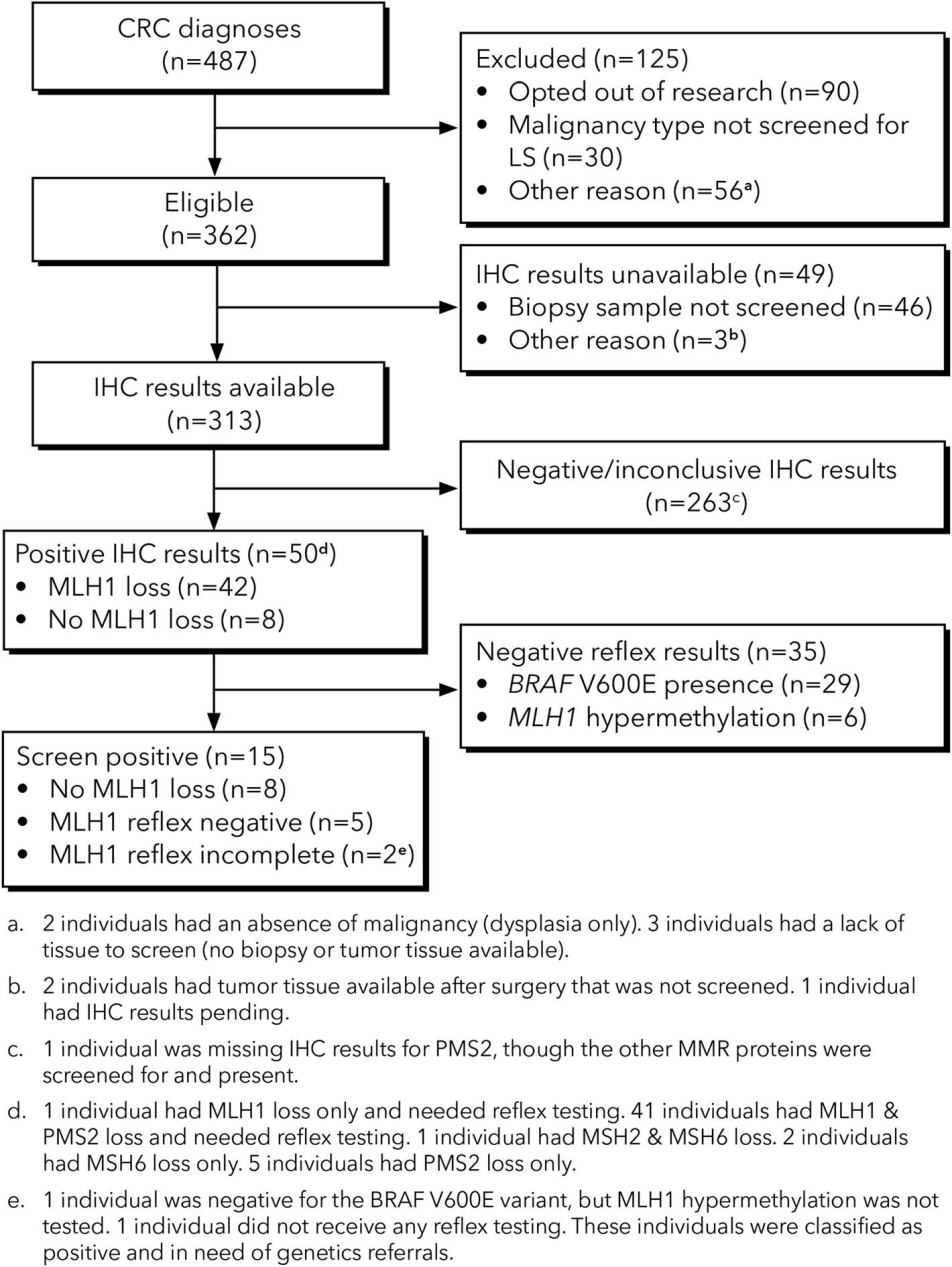
UTS results in individuals newly diagnosed with CRC (Jan. 1, 2016 – Jul. 31, 2018)

**Fig. 2:**
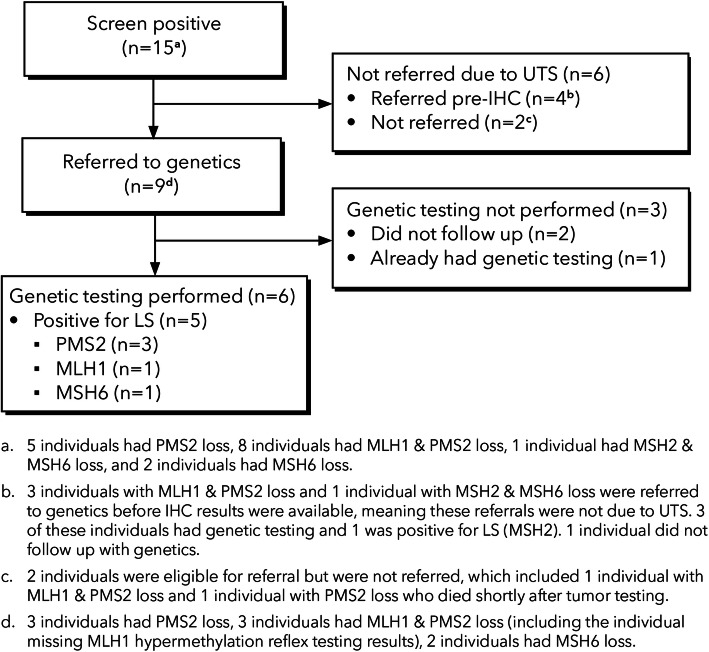
Genetic referrals and follow-up among the 15 individuals diagnosed with CRC with a positive tumor screen who screened positive (Jan. 1, 2016 - Jul. 31, 2018) [Continuation from the last box at the bottom of Fig. 1]

The screening and referral process of patients diagnosed with CRC are summarized in Figs. 1 and 2.

Of the 362 individuals eligible for UTS, 49 were not screened by IHC. Most of the individuals not screened by IHC (46/49 or 94%) only had biopsy samples available. However, 78 of the 313 who were screened by IHC also only had a biopsy sample available. This indicates there was inconsistent use of biopsy samples for screening by IHC when tumor resections were not available.

Overall, among the nine individuals who were referred to genetics after screening positive for LS, seven (78%) followed up with genetics. Of the seven individuals that followed up with genetics after a referral based on IHC results, six had germline genetic testing for LS, and one individual who was found to have received a diagnosis of LS previously was not retested. Five (83%) of the six individuals who had germline genetic testing for LS had a subsequent diagnosis of LS. One additional individual was diagnosed with LS from the four individuals who screened positive but were referred prior to IHC results. These four individuals would have been eligible for referral due to UTS results had they not already been referred. Of those with a diagnosis of LS, age at onset of cancer ranged from 32 to 81 years.

Of the 262 individuals that had a negative screening result (i.e., all 4 MMR proteins were present on IHC), 44 were referred for to genetics although it was not indicated by UTS (24 prior to UTS and 20 after UTS results were available). In addition, of the 35 who showed loss of at least one MMR proteins by IHC but had *BRAF* V600E or *MLH1* hypermethylation present, 10 were referred to genetics (four prior to UTS and six after UTS results were available). Of these 54 referred to genetics, 32 (59%) had a visit with genetics and 24 (44%) underwent germline genetic testing for LS. Only one of the individuals who underwent genetic testing (4%) was diagnosed with LS; the individual had loss of MLH1 on IHC and *BRAF* V600E was present in reflex testing but had been referred to genetic testing prior to IHC and was found to have a pathogenic variant in *MSH6*). Because of the inconsistencies between this individual’s LS diagnosis, the IHC, and reflex results, it is suspected that there was an error in IHC results. Laboratory services declined a request for repeat testing to follow up on this inconsistency. A variant of unknown significance (VUS) in *MSH2* was detected in a second case (no MMR protein loss on IHC).

Among the 313 individuals with CRC with IHC results, 15 cases received a positive screen on IHC and were negative for *BRAF* V600E and *MLH1* hypermethylation. Among these 15 cases, 6 underwent genetic testing and were diagnosed with LS giving a detection rate of 6/313 or 1.9% (95% CI: 0.88 − 4.12%) (see Table 1 for characteristics of these patients). This estimate focuses on the diagnosis of LS in patients with CRC who underwent genetic testing in conjunction with the current tumor screen and does not include two LS cases noted above: (1) the individual with a positive IHC screen who had received a prior diagnosis of LS and did not undergo genetic testing again and (2) the individual who had a positive IHC screen and BRAF V600E was present on reflex testing but was diagnosed with LS following genetic testing. The detection rate is significantly greater than the proportion of LS expected in the general population (1/279 or 0.35%), yet it is less than the expected proportion among CRC patients (3%). The detection rate is an underestimate of the sample LS prevalence.

**Table 1:** Characteristics of Individuals Diagnosed with LS from UTS or Referral to Genetics after a CRC Diagnosis

Case	Age at CRC Diagnosis	MMR Gene	Bethesda Guidelines	Amsterdam II Criteria
1	73	*MLH1*	Yes	No
2*	32	*MSH2*	Yes	Yes
3	81	*MSH6*	No	Yes
4	32	*PMS2*	Yes	No
5	69	*PMS2*	No	No
6	57	*PMS2*	Inconclusive	No

* Case 2 was referred to genetics before IHC results from UTS were completed. All other individuals were referred after IHC from UTS was completed.

### Universal tumor screening of EC cases

**Fig. 3:**
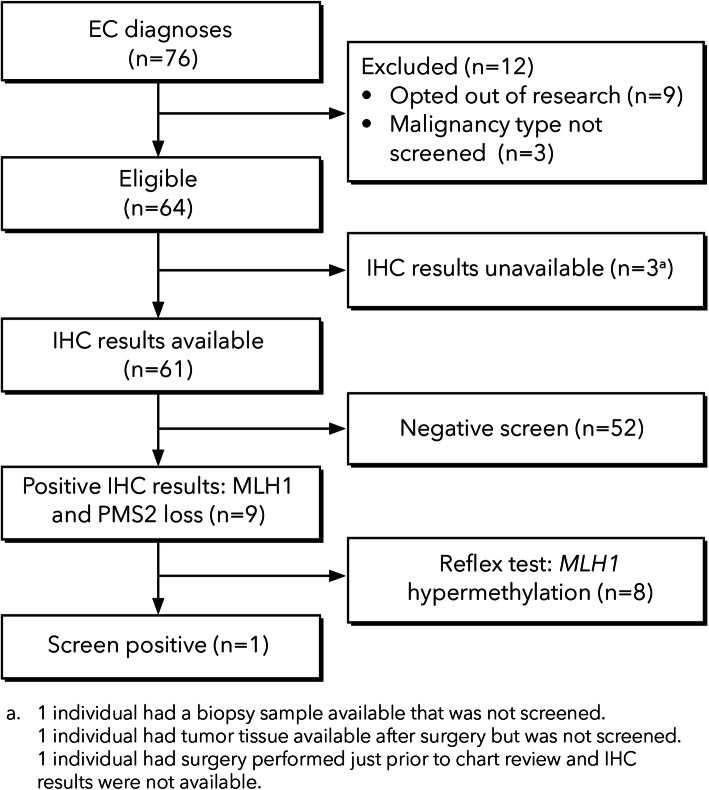
UTS and genetic referrals for individuals newly diagnosed with EC (Nov. 1, 2016 - Jul. 21, 2018)

The screening and referral process of patients diagnosed with EC is summarized in Fig. 3.

One individual was eligible for referral for genetic evaluation based on a positive screen for LS. This individual was referred to genetics after IHC results were available. The patient underwent genetic testing which was negative for LS.

One individual was not screened although a biopsy sample was available. Biopsy samples were used for three of the individuals with IHC results available.

Of the 52 individuals that had a negative result for IHC (i.e., all four MMR proteins were present), seven were referred to genetics (four prior to IHC results and three after IHC results were available). In addition, of the eight who had a positive screen by IHC but had *MLH1* hypermethylation present, five were referred to genetics (all after IHC results were available). Of these 12 referred to genetics, nine had a visit with genetics and four underwent germline genetic testing for LS. None of these individuals were diagnosed with LS. One individual with a negative screen on IHC who was referred to and had a visit with genetics was not retested; prior genetic testing was negative for LS but did detect two *MUTYH* pathogenic variants.

## Discussion

This study assesses the effectiveness of a UTS for LS implemented in an integrated healthcare system. The UTS program contributed to the identification of 5 cases of LS out of the 426 individuals who were eligible for screening by IHC. These individuals may not have received a referral to genetics without screening positive. In addition, there was a high frequency of follow-up for individuals (80%) that screened positive and were referred to genetics, which has been reported as a considerable barrier to screening success in other health systems [40, 41].

However, opportunities for improvement were identified through this analysis. During the study period, tumor tissue resections were used for screening at KPNW, while biopsy samples are inconsistently used. In 78 of the 313 CRC patients screened by IHC, biopsy or polypectomy samples were used. In 3 of the 61 EC patients screened by IHC, biopsy samples were used. Consistent use of biopsy samples could have reduced the number of individuals who had tissue available but were not screened by IHC from 52 to as few as five individuals. The use of biopsy samples for UTS is being explored by KPNW as a result. Several studies have found comparable MMR when comparing biopsy samples to tissue resections in both EC and CRC patients [42–46]. There is also evidence to support the use of biopsy samples for *BRAF* V600E testing, a part of the reflex testing for CRC patients showing a loss of MLH1 [47–49]. Using biopsy samples would also allow for LS diagnosis before surgical treatment. Knowledge of LS status could alter decision making for surgical treatment of tumors. The US Multi-Society Task Force on Colorectal Cancer (MSTF) published a set of guidelines on LS management which recommends the use of biopsy samples for this reason. [33] Extended, subtotal, or total colectomies, for example, may be recommended for patients with LS [50, 51]. Preoperative LS diagnosis would allow patients to be fully informed about their own risks and recommended surgical treatment options.

In addition to individuals with biopsy samples available that were not screened by IHC, other missed opportunities included individuals with tumor tissue available that were not screened by IHC and individuals who screened positive that did not end up being referred to genetics. Some individuals diagnosed with CRC and EC were under the age of 50 but had a normal screening result and were not referred to genetics. UTS may not correctly identify every individual who is affected by LS [52]. For example, one individual was found to have an *MSH6* pathogenic variant because they were referred to genetics and genetic testing was ordered prior to the completion of IHC screening. In this case, there were inconsistencies with the screening results and genetic findings that are unresolved, as IHC noted a loss of MLH1 and reflex testing noted the presence of *BRAF* V600E which led to a false negative screening result. Individuals meeting other high-risk criteria, such as those with a young age of onset, may still benefit from genetic counseling for hereditary colorectal cancer. The benefit of genetic testing in this group should be further explored.

Three of the 5 cases (60%) of individuals diagnosed with LS following UTS had a pathogenic variant in *PMS2*. These results are similar to a prior study at KPNW that assessed the feasibility of a UTS program among newly diagnosed cases of CRC prior to the implementation of the UTS program. [53] The results of this prior study indicated that a higher proportion of cases diagnosed with LS after being offered tumor screening for LS had a pathogenic variant in *PMS2* compared to individuals who were diagnosed with LS after being referred for genetic counseling (selective screening) (50% vs. 22%, respectively). Given *PMS2* variants are associated with a lower penetrance and older age of onset compared to other genes associated with LS, it is most likely that they are not referred for genetic counseling based on selective screening because they do not meet high-risk criteria and may be more likely to be identified by UTS programs.

Limitations to this study include small sample sizes. Specifically, detection rates had a wide margin of error due to the limited sample size. In addition, we expect UTS for LS in the individuals diagnosed with EC to be effective at identifying LS. However, in part due to the limited number of individuals included in our analysis, the UTS program did not identify any cases of LS in this group. Similar integrated health care systems have reported UTS to be ineffective at increasing LS detection in EC patients. [54] More work should be done to explore this area. Other limitations include challenges with generalizability: KPNW is a single, integrated health care system with patients who have health insurance and reside in/around Oregon and southwest Washington.

Diagnosing LS through UTS at KPNW was one step in addressing the low proportion of individuals who are aware of their increased cancer risk. This program directly led to diagnoses for 5 individuals. There is also potential to indirectly lead to further diagnoses through cascade screening and testing: the identification and genetic testing of at-risk family members. Other health systems looking to improve LS identification in their patient population will likely encounter similar challenges to those described in this paper. It is our hope that sharing the experiences and lessons learned at KPNW may assist others during implementation or quality improvement of UTS for LS.

### Conclusions

We performed a systematic assessment of a program that screens all newly diagnosed cases of CRC and EC for Lynch syndrome in an integrated healthcare setting. This study found high rates of follow-up for confirmatory germline testing for pathogenic variants associated with LS among patients who screened positive. In addition, we identified ways that the program could be improved, such as consistent use of biopsy samples for screening. Overall, these findings can be used to provide guidance to other healthcare systems to improve or implement such screening programs.

## Data Availability

Reasonable requests for the datasets generated and/or analyzed during the current study can be sent to the corresponding author.

## Abbreviations

CRC: Colorectal cancer
EC: Endometrial cancer
EMR: Electronic medical record
IHC: Immunohistochemistry
KPNW: Kaiser Permanente Northwest
LS: Lynch syndrome
MMR: Mismatch repair
MSI: Microsatellite instability
MSTF: US Multi-Society Task Force on Colorectal Cancer
UTS: Universal tumor screening
VUS: Variant of uncertain significance
